# SpQuant-SNN: ultra-low precision membrane potential with sparse activations unlock the potential of on-device spiking neural networks applications

**DOI:** 10.3389/fnins.2024.1440000

**Published:** 2024-09-04

**Authors:** Ahmed Hasssan, Jian Meng, Anupreetham Anupreetham, Jae-sun Seo

**Affiliations:** School of Electrical and Computer Engineering, Cornell Tech, New York, NY, United States

**Keywords:** spiking neural networks, quantization, pruning, event data, static images, low-precision, membrane potential, leaky

## Abstract

Spiking neural networks (SNNs) have received increasing attention due to their high biological plausibility and energy efficiency. The binary spike-based information propagation enables efficient sparse computation in event-based and static computer vision applications. However, the weight precision and especially the membrane potential precision remain as high-precision values (e.g., 32 bits) in state-of-the-art SNN algorithms. Each neuron in an SNN stores the membrane potential over time and typically updates its value in every time step. Such frequent read/write operations of high-precision membrane potential incur storage and memory access overhead in SNNs, which undermines the SNNs' compatibility with resource-constrained hardware. To resolve this inefficiency, prior works have explored the time step reduction and low-precision representation of membrane potential at a limited scale and reported significant accuracy drops. Furthermore, while recent advances in on-device AI present pruning and quantization optimization with different architectures and datasets, simultaneous pruning with quantization is highly under-explored in SNNs. In this work, we present *SpQuant-SNN*, a fully-quantized spiking neural network with *ultra-low precision weights, membrane potential, and high spatial-channel sparsity*, enabling the end-to-end low precision with significantly reduced operations on SNN. First, we propose an integer-only quantization scheme for the membrane potential with a stacked surrogate gradient function, a simple-yet-effective method that enables the smooth learning process of quantized SNN training. Second, we implement spatial-channel pruning with membrane potential prior, toward reducing the layer-wise computational complexity, and floating-point operations (FLOPs) in SNNs. Finally, to further improve the accuracy of low-precision and sparse SNN, we propose a self-adaptive learnable potential threshold for SNN training. Equipped with high biological adaptiveness, minimal computations, and memory utilization, SpQuant-SNN achieves state-of-the-art performance across multiple SNN models for both event-based and static image datasets, including both image classification and object detection tasks. The proposed SpQuant-SNN achieved up to 13× memory reduction and >4.7× FLOPs reduction with < 1.8% accuracy degradation for both classification and object detection tasks, compared to the SOTA baseline.

## 1 Introduction

In the biological nervous system, cortex neurons convert varied inputs into electrical signals or spikes. Spiking Neural Networks (SNNs) mimic this by processing the inputs over time, with gradual increments in their internal energy. Neuronal behavior of leaky integration and fire (LIF) in SNNs accumulates membrane potential over time and produces spikes for the membrane values exceeding the potential threshold. This leads to an efficient information encoding method with binary spikes (0 or 1). Such spatial-temporal computation promotes SNN as an attractive AI solution with both biological plausibility and energy efficiency in comparison to the conventional artificial neural networks (ANNs) (He et al., 2016). Moreover, layer-by-layer information processing using binary spikes benefits cognitive processing on edge devices, where stringent power and area requirements are posed. Furthermore, latency-sensitive computer vision tasks such as efficient detection and tracking of fast-moving objects require end-to-end sparse and energy-efficient computational flow. Event-based cameras or Dynamic Vision Sensors (DVS) (Gallego et al., 2020) provide a binary input stream that directly connects with SNNs to serve rapid object tracing. Binarized spatial-temporal data alignment with Spiking Neural Networks (SNNs), shrinks the gap between computer vision and neuromorphic computing.

Despite such benefits, the direct training process of SNN is challenging due to the non-differentiability of the spike function. Early researchers relied on the ANN-to-SNN conversion (Diehl et al., 2015; Han et al., 2020) to train SNN models with additional training iterations. These approaches fail to achieve sufficiently high accuracy with extra computations. Subsequently, various direct training methods have been proposed to improve the accuracy of SNNs using surrogate gradient (SG) functions (Lee et al., 2016; Wu et al., 2019; Deng et al., 2021), which approximate and propagate the gradient during learning. However, the inaccurate approximation and heuristic SG selection hurt the training stability of deep SNN models, which further motivated the temporal normalization method (Zheng et al., 2021) and output regularization techniques (Deng et al., 2021; Guo et al., 2022) to smooth the loss.

**Figure 1:**
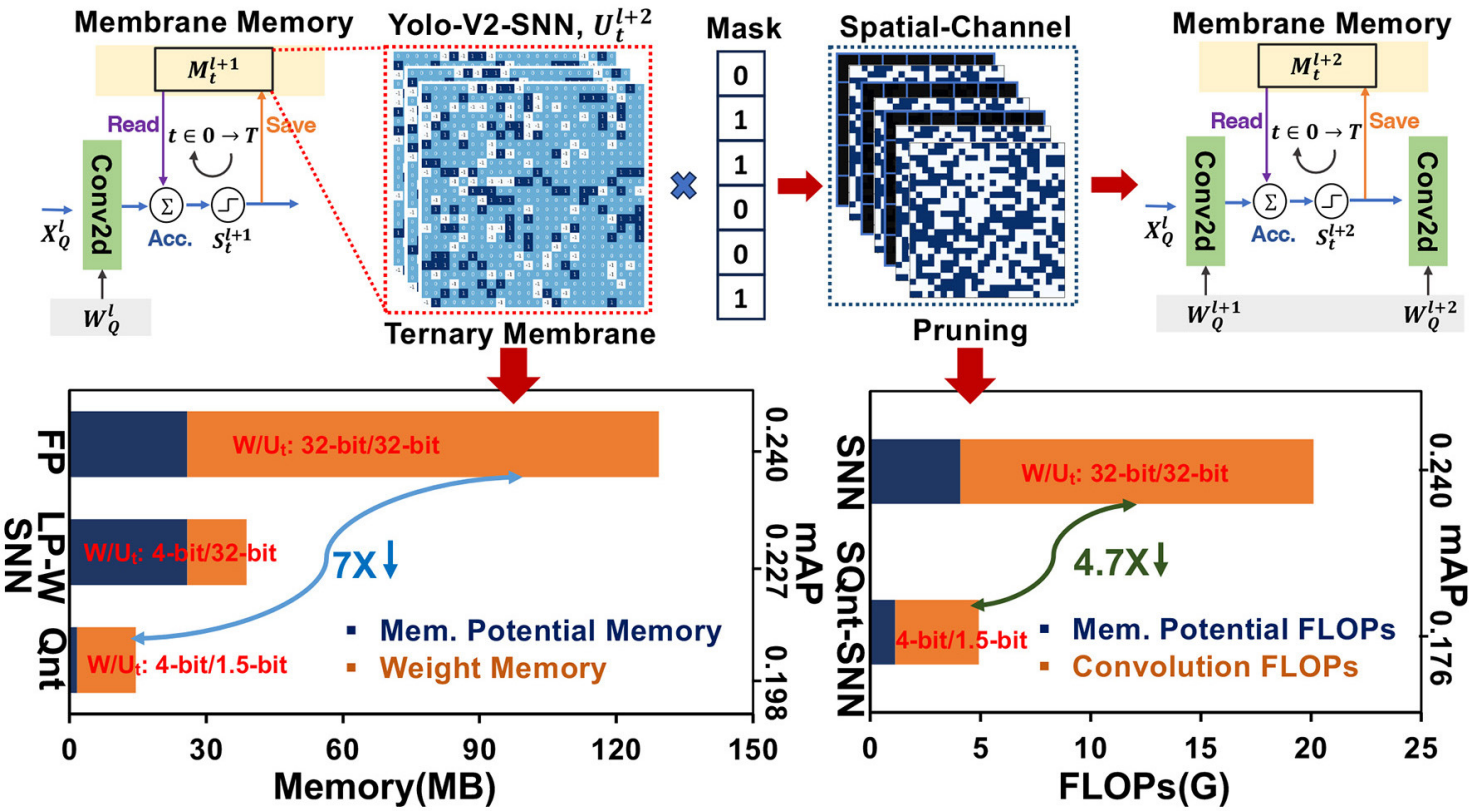
Memory cost and FLOPs reduction of proposed SpQuant-SNN with YOLO-V2 on Prophesse Gen 1.

Most of these SNN algorithms have focused on achieving high accuracy while employing full precision (FP32) weights and membrane potential (Deng et al., 2021; Li et al., 2021b; Meng et al., 2022). Despite the binary information propagation in event-based SNNs, membrane potential accumulation and weight updates employ high-precision computation. The membrane potential values for every neuron must be stored and updated in the local memory for consecutive time steps. This leads to cyclic memory access during the read-modify-write process of membrane potential over multiple time steps. Since the average firing rate in SNNs stays low, the post-spike membrane potential saving for high-resolution images requires significantly high memory with extra redundant computations. Additionally, membrane potential shows long tails of neurons that are less likely to fire for limited time steps. These inactive neurons utilize excessive local memory and dynamic energy that hurts the hardware-level computational efficiency in deep SNNs. In Figure 1, we profile the weight and membrane potential memory along with convolution and membrane potential FLOPs of SNN-Yolov2 architecture on the Prophesee Gen1 dataset. In comparison to full-precision SNN-Yolov2 baseline (FP), 4-bit weight quantization only (LP-W) reduces the weight memory but the full-precision membrane potential memory dominates in this situation. However, quantizing both weights and membrane potential in SNN-Yolov2 (Qnt) reduces the overall memory by >7 ×. Similarly, exploring the pruning opportunities in quantized-SNN-Yolov2 (SpQuant-SNN) can help reduce the FLOPs by >4.7×.

To address these memory computation issues, some recent works investigate the low-precision representation and pruning in SNNs at a limited scale. Chowdhury et al. (2021) and Schaefer et al. (2023) have used 2-bit precision and 5-bit precision of weights to reduce the weight memory footprint, but these works use FP32 precision for the membrane potential. In addition, Chowdhury et al. (2021) used time step-based pruning to reduce the temporal computations at the cost of lower inference accuracy. Loihi (Davies et al., 2018) have used 12-bit and 24-bit fixed-point membrane potential representation with low-precision weights to optimize the SNN hardware resources. However, to support such high precision (12-bit and 24-bit) of membrane potential with high-resolution input data is challenging on edge devices due to extra memory requirements. A dense feature map in the shape of membrane potential doubles the computation cost by demanding high energy during accumulate and fire operations. Furthermore, Loihi (Davies et al., 2018) does not report the memory vs accuracy and energy vs. accuracy trade-off against high-resolution datasets, i. e. ImageNet-100 and ImageNet-1k. Q-SpiSNN (Putra et al., 2021) implemented low-precision membrane potential and weight to showcase its performance using the relatively simpler MNIST and DVS-CIFAR10 datasets. Apart from quantization, some prior works (Perez-Nieves and Goodman, 2021; Lien and Chang, 2022) explored pruning opportunities in SNNs to skip the computation by masking out the negative membrane potential. Kim et al. (2022) proposes a lottery ticket hypothesis to reduce the time steps and active weights by applying a pruned weight mask in SNN. Although these works achieve high feature-level compression, the memory consumption due to the full precision of membrane potential is significantly prominent. Disjoint quantization and pruning in prior SNN works fail to achieve minimum memory consumption and number of operations with high accuracy.

To bridge these research gaps, we propose *SpQuant-SNN*, a fully-quantized spiking neural network with *ultra-low precision weights, membrane potential, and high spatial-channel sparsity*, enabling the end-to-end low precision with significantly reduced operations on SNN. SpQuant-SNN achieves outstanding memory, and energy efficiency with negligible accuracy degradation compared to the SOTA SNN baseline. To further improve the performance, we incorporate a novel self-adaptive SNN training algorithm with the learnable threshold to improve the adaptability of SpQuant-SNN. The proposed SpQuant-SNN makes the following key contributions to advance SNN performance for software hardware co-design.

- We propose a novel quantization-aware training algorithm designed for integer-only SNN. In particular, we propose Stacked Gradient Surrogation (SGS), a novel SNN training scheme designed for low-precision membrane potential training. As a result, SpQuant-SNN achieves up to 7.01 × and 13 × total inference memory reduction on complex static image and event datasets with deep SNN architectures.
- We present a novel membrane potential aware spatial-channel dynamic pruning method to reduce the FLOPs without losing high performance. SpQuant-SNN achieves >4.7 × FLOPs reduction from the baseline with 80% sparsity on complex static image and event datasets with deep SNN architectures.
- We propose a layer-wise learnable threshold scheme with threshold optimization method to improve the training stability and enhance the adaptability of SNNs. With the learnable threshold, SpQuant-SNN performance is improved by >1.58% and 0.07 in terms of inference accuracy and mAP value respectively.

## 2 Related work

### 2.1 ANN to SNN conversion

Early research works (Diehl et al., 2015; Rueckauer et al., 2016) converted a high-performance non-spiking ANN model into a spiking version to resolve the non-differentiability issues. This conversion-based method relies on the fact that the SNN firing rate can be estimated by ANN activation for a corresponding architecture. ANN-to-SNN conversion helps determine SNN parameters directly from an ANN model without losing significant performance (Meng et al., 2022). Some of the prior works incorporate further optimizations including weight normalization (Sengupta et al., 2019), temporal switch encoding (Han and Roy, 2020), rate norm layer (Ding et al., 2021), and bias shift (Deng and Gu, 2021) to match the converted SNN and ANN performance. However, these approaches require more training time, high computation overhead, and additional efforts for the overall training. Several methods have been proposed to improve the latency of the converted model by tuning different parameters (Han et al., 2020) and quantizing the model weights (Li et al., 2022) to compensate for the over-training cost but these approaches fail to match the performance of converted SNN to high accuracy of direct SNN training methods.

### 2.2 Direct SNN training

Most of the SNN training works use gradient approximations to resolve non-differentiability issue for direct training of SNNs. BNTT (Neftci et al., 2019) introduces the gradient surrogation, which approximates the gradient landscape by the designed non-linear function (e.g., Sigmoid). To improve the training stability and accuracy, various SG functions have been implemented in the literature, such as rectangle function, arctangent, and triangle functions (Deng et al., 2021). Similarly, DSpike (Cannici et al., 2020) implements a non-linear function in the forward pass during SNN training. In the meantime, the emergence of the temporal-batch normalization (Zheng et al., 2021) and residual gradient paths enables stable SNN training with deep models. Additionally, some recent works introduce the temporal gradient approximation (Shen et al., 2022), where the spatial-temporal computation of SNN can be considered as a special version of a recurrent neural network (RNN) (Meng et al., 2022; Shen et al., 2022). These emerging trends in direct SNN training introduce spike-based computer vision using large and deep architectures (Zhou et al., 2023a) with large-scale datasets (e.g., ImageNet).

### 2.3 Quantization of SNN

Motivated by the nature of SNN with binary spikes, prior works have investigated low-precision SNN. Chowdhury et al. (2021) uses post-training quantization (PTQ) to compress the low-precision weights down to 5-bit for inference. Another work (Li et al., 2022) demonstrates weight quantization using ANN-SNN conversion approach with ImageNet dataset. Quantization of convolution output and weights is also explored in one of the recent works to reduce the memory footprint (Castagnetti et al., 2023). Since SNN introduces the temporal dimension during inference, besides quantizing the weights (Davies et al., 2018; Yin et al., 2023), considering the quantization of membrane potential is important for memory-efficient SNNs Figure 1. Some recent works have explored membrane potential quantization in SNNs, albeit to a limited scale with a significant performance drop. Q-SpiNN (Putra et al., 2021) uses PTQ and QAT for low-precision representation of membrane potential and weights at a limited scale. Using 4-bit precision of weight and membrane potential, the proposed work reports a significant drop in accuracy for MNIST and DVS-Gensture datasets. Furthermore, Putra et al. (2022) has also employed fixed-point representation for the membrane potential. However, none of these methods are verified against large-scale datasets or achieved the ultra-low-precision (≤ 4-bit) membrane. Additionally, the naive implementation of conventional symmetric or asymmetric quantization decreases the hardware benefits of SNNs. The accumulation of membrane potential across multiple time steps makes the hardware-aware quantization process challenging.

#### 2.3.1 Optimal quantization boundary selection

The key aspect of the quantization algorithm involves determining the optimal clipping boundary within the full-precision range of weights and activation in the deep neural network (DNN). However, Spiking Neural Networks (SNNs) are different because the membrane potential is iteratively updated at various time steps during inference. Thus, identifying the ideal clipping boundary is critical and challenging for the effective quantization of the membrane potential in SNNs.

#### 2.3.2 Incompatibility of iterative dequantization in SNNs

The standard quantization process, involving the high-precision scaling factor, includes the “quantize and dequantize” workflow to scale the low-precision representation (e.g., INT8) back to the high precision floating point range. As demonstrated by the prior work (Jacob et al., 2018), post-quantization scaling is required to avoid the mismatched numerical range. In SNN, time step information requires iterative quantization to maintain low precision. However, rescaling the updated membrane potential at **each** time step magnifies the cost of its hardware implementation. Therefore, the traditional quantization-aware training (QAT) scheme is incompatible with low-precision membrane potential. Furthermore, the choice of integer-only representation of membrane potential gives infinite value in the backward propagation. The cost of conventional QAT scaling at the hardware level and the non-differentiability of integer-only quantization, make the low-precision representation of the membrane challenging.

### 2.4 Pruning of SNN

Different from the static weight pruning in DNNs, sparsity in SNN can be explored in the weights and time domain. Apart from quantization, recent works (Perez-Nieves and Goodman, 2021; Lien and Chang, 2022) have explored sparse SNN training to compress the models' computations with reduced computations. Kim et al. (2022) investigated the lottery ticket hypothesis to SNNs, which explores the winning ticket in both weights and temporal steps for the computation skipping. However, most of these previous works have explored pruning and quantization dis-jointly with significant performance degradation on simple datasets.

#### 2.4.1 Quantized SNNs with pruning opportunities

Some of the prior works have tried to explore SNN pruning with quantization using naive DNN-based approaches. Chowdhury et al. (2021) jointly compresses the low-precision weights down to 5-bit and sparsify the SNN with temporal pruning during training. Another work investigates NM-sparsity (Chen et al., 2018) by masking the negative membrane potential values in the spatial domain. Most of these works have either implemented naive masking on negative neuron values or introduced extra computations for weight or activation skipping. Furthermore, the quantized membrane potential tensors, in these cases are floating point values that incur high-precision scaling costs during the cyclic quantization and dequantization process. In addition, temporal pruning without considering membrane potential saliency degrades SNN performance with complex datasets.

### 2.5 Learnable dynamics in SNN

SNN involves parametrized neurons (e.g., LIF neurons) with spike functions. Instead of heuristic parameter selection, very limited prior works consider trainable optimization of spike neurons. Neuroscience work (Kole and Stuart, 2008) with the location-dependent potential threshold in nervous systems implies the adaptive firing procedure within the mechanism of spike generation. Following this, some recent works introduced the learning dynamics into SNN training, albeit to a limited degree. Fang et al. (2021) uses a large-sized SNN model and extensive training efforts (up to 1,024 epochs) to introduce the learnable time constant for direct SNN training. LTMD (Wang et al., 2022) introduced the learnable neuron threshold with dropout in SNN training. Optimization of threshold value based on naive weight gradient in the backward pass compromised LTMD performance against basic event datasets. Similarly, DSR (Meng et al., 2022) optimizes the potential threshold during training by multiplying a scaling factor α. In addition, the binary output spikes generated by DSR are multiplied by the threshold value. Iterative high-precision scaling with a fixed ratio limits the adaptiveness, resource efficiency, and freedom of SNN learning and makes it computationally inefficient for resource-constrained hardware.

## 3 Basics of spiking neural networks

SNNs mimic the biological nervous system and propagate binary spikes in the spatial-temporal domain. Membrane potential exceeding the threshold value in the Leaky Integrate-and-Fire (LIF) function generates spikes, Equation 1.


(1) $$\begin{array}{l} u_{t}^{l}=\tau u_{t-1}^{l}\left(1-S_{t-1}^{l}\right)+I_{t}^{l}\;\text{and }I_{t}^{l}=\underset{i}{\sum }w_{i}^{l}S_{i}^{l} \end{array}$$

Where *u*_*t*_ represents the membrane potential for layer index *l* at time *t*, $S_{t-1}^{l}$ is the output spike of the previous time step, and $I_{t}^{l}$ is the synapse current for layer index *l* at time *t*, *w* is the synapse weight, and τ is the time constant. In our experiments, we set τ = 0.5.

During the **forward pass**, the membrane potential is accumulated with high precision and if it exceeds the potential threshold value, the spike is generated according to Equation 2.


(2) $$S_{t}^{l}=\theta (u_{t}^{l}-V_{th})=\{\begin{array}{ll} 1 & \text{ if }u_{t}^{l}\geq V_{th}\\ 0 & \text{ otherwise} \end{array}$$

Where θ represents the Heaviside step function, and *V*_*th*_ represents the membrane potential threshold for spiking neurons. In the **backward pass**, the weight gradient can be computed based on Equation 3:


(3) $$\begin{array}{l} \frac{\partial L}{\partial W^{l}}=\underset{t}{\sum }\frac{\partial L}{\partial S_{t}^{l}}\frac{\partial S_{t}^{l}}{\partial u_{t}^{l}}\frac{\partial u_{t}^{l}}{\partial I_{t}^{l}}\frac{\partial I_{t}^{l}}{\partial W^{l}} \end{array}$$

Since the Heaviside step function is non-differentiable in nature, $\frac{\partial S_{t}}{\partial u_{t}}$ becomes non-deterministic. To continue the learnability in training, backward pass approximations are implemented in SNNs. Various surrogate gradient functions are proposed to preserve the differentiability in SNNs (Lee et al., 2016; Wu et al., 2019; Che et al., 2022; Chen et al., 2022) and we adopt vanilla triangle function as mentioned in Equation 4:


(4) $$\begin{array}{l} \frac{\partial S_{t}}{\partial u_{t}^{l}}=\theta^{\prime }\left(u_{t}^{l}-V_{th}\right)=\max\left(0,1-|u_{t}^{l}-V_{th}|\right) \end{array}$$

## 4 Proposed method

Full-precision SNNs without pruning are hard to implement on the resource-constrained hardware. To achieve maximum hardware awareness with high performance, we sequentially implement low-precision, sparsity, and adaptability in SNN. Starting from membrane potential and weight quantization for low-precision SNN (Quant-SNN), we explore and resolve the challenges of clipping boundary selection and interactive scaling during the quantization process in Section 4.1 and Section 4.2. Further, we analyze the abundance of negative membrane potential with a low-spiking rate in Quant-SNN and propose a solution to explore the pruning opportunities by implementing sparse and quantized SNN (SpQuant-SNN) in Section 4.3. Finally, we discuss the gradient mismatch dynamics in SNN and implement a layer-wise adaptive threshold to improve the performance of SpQuant-SNN in Section 4.4.

### 4.1 Membrane potential clipping for deterministic boundary

In SNNs, post-spike membrane potentials are stored in the local memory and get iteratively fetched for accumulation in the next time steps. To maintain low precision of membrane potential, time step information requires iterative quantization. Clipping boundary selection during the quantization process for **each** time step elevates the cost of its hardware deployment. To address this challenge, we aim to unify the negative clipping boundary of membrane potential for each time step. We propose a two-step analysis for evaluating the robustness of SNN with respect to different quantization boundaries.

We first denote the negative clipping boundary as *c*, and the entire quantization range becomes [*c*, γ], where γ is the maximum membrane potential after spiking. Naturally, 0 ≤ γ < *V*_*th*_. The mechanism of “accumulate-and-fire” of SNN makes each membrane potential neuron possible to fire during the consecutive time steps. However, the relationship between membrane potential value and spiking activity is non-observable during inference. Let's assume that **Γ**_*u*_ represents the membrane values that are below the clipping threshold *c*. Naively unifying all the **Γ**_*u*_ values to the clipping threshold *c* will change the spiking rate of the next time step, and also the final output of the layer. To quantify such impact, we investigate the robustness of SNN with respect to different clipping thresholds *c* with the following two perspectives:

- **Step 1:** We first quantify the impact of the membrane potential clipping by analyzing the firing rate of neurons **Γ**_*u*_.
- **Step 2:** We evaluate the robustness and accuracy of the SNN model with different clipping thresholds.

Given the input sample *X* of the size of *N*×*T*×*C*×*H*×*W*, with total time steps = *T*, we first investigate the distribution of the post-spike membrane potential ${û}_{t}^{l}$ at the very first time step *t* = 1. Mathematically, the membrane values **Γ**_*u*_ are defined as below in Equation 5:


(5) $$\begin{array}{l} \Gamma_{u}=\left\{{û}_{t=1}^{l}|{û}_{t=1}^{l}<c\right\} \end{array}$$

For the remaining *t*∈[2, …*T*] time steps, the firing rate *r* of **Γ**_*u*_ can be computed using Equation 6:


(6) $$\begin{array}{l} r_{s}=\frac{1}{T}\underset{t}{\sum }\frac{\text{card}\left(M_{t}\right)}{C\times H\times W}\;\text{where}M_{t}=1_{\Gamma_{\text{u}}}\left({\text{u}}^{\text{l}}\right)\wedge {\text{S}}_{\text{t}}^{\text{l}} \end{array}$$

Where 1 is the indicator function and it returns the binary flag that represents whether the membrane value *u* is unfired and also small enough. ∧ is the “AND” logic, $S_{t}^{l}$ is the binary spikes of layer *l* at time step *t*, and card(·) returns the number of non-zero elements inside M. Therefore, *r*_*s*_ in Equation 6 characterizes **how many membrane potential neuron values are initially (*****t*** **= 1) silent and inactive (less than** ***c*****) but spike in the future** ***t***.

**Figure 2:**
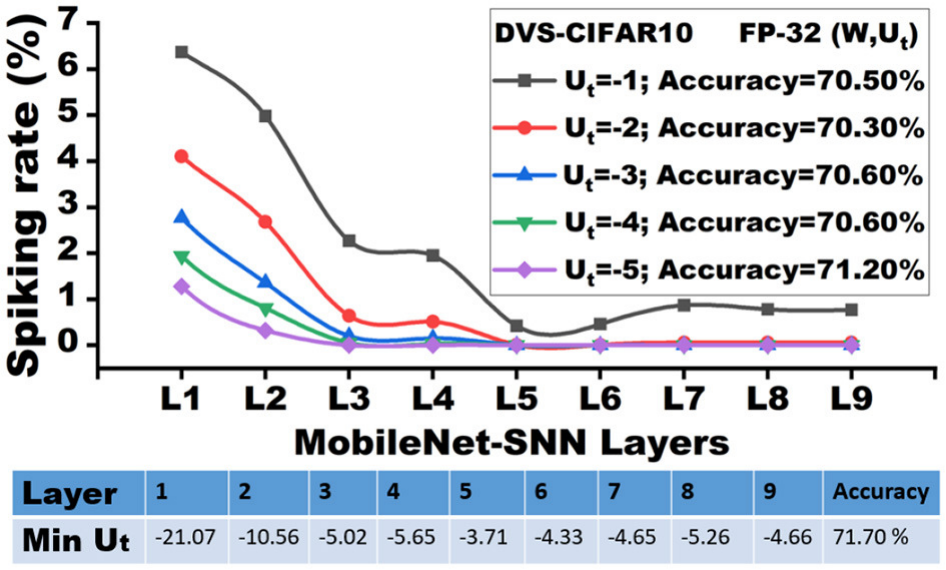
Analysis of spiking activity with different membrane potential clipping boundaries.

Based on the theoretical setup above, we sweep over different clipping boundaries *c* from -5.0 to -1.0. For each value of *c*, we compute the average firing rate *r*_*s*_ across each *t*∈[2…*T*], and record the layer-wise firing rate (percentage). Figure 2 shows the layer-wise firing rate of a 9-layer lightweight MobileNet-V1 SNN pre-trained on the DVS-CIFAR10 dataset with 30 total time steps. Compared to the widely used ResNet models, the lightweight MobileNet models exhibit higher sensitivity to quantization (Park and Yoo, 2020), which provides accurate insights into the clipping distortion.

Let's assume the minimum membrane potential (Min *U*_*t*_) of each layer is *U*_min_. As shown in Figure 2, the early layers exhibit a high magnitude of *U*_min_ (e.g., -21.07 for the first layer) in the floating point baseline. However, there are only 6.35% of neurons that fire in the future time steps with all the accumulations, where *U*_min_<*U*_*t*_ < −1. In other words, the remaining unfired 93.6% of neurons create a “silent region”, where the membrane neurons have **zero contribution** to future time steps and the next layer. Although *U*_min_ increases in the latter layers, the *silent region* in the negative membrane potential extends. As a result, clipping distortions have a negligible impact on the majority of the membrane potential, even with the most aggressive clipping (c=-1.0). Therefore, we have the following observation:

**Observation**: *If the membrane potentials are negative enough, their impact on spikes is minimal*.

We further prove the clipping robustness with the accuracy impact during inference with no fine-tuning.

Figure 2 shows that, given the pre-trained full-precision model with 71.70% inference accuracy for DVSCIFAR-10 dataset, when the membrane potential is clipped to *c* = −1.0, the inference accuracy can still be maintained at 70.50%. We extend the verification of this observation on CIFAR-10 dataset with ResNet-19 and validate the accuracy degradation by sweeping the clipping boundary values from -5.0 to -1.0, as reported in Table 1. The minimal accuracy degradation allows us to extend the **Observation** into: *If the membrane potential values are negative enough, their impact on accuracy is minimal*, ***which implies the robustness of membrane potential to low-precision*** ***representation***.

**Table 1:** Robustness of Quant-SNN for ResNet-19 against different negative clipping boundaries.

**Clipping threshold**	FP-Baseline	−5.0	−4.0	−3.0	−2.0	−1.0
**CIFAR-10 Accuracy**	94.53	94.33	94.21	94.34	94.22	94.09

### 4.2 Quantization algorithm

#### 4.2.1 Incorporating membrane potential quantization in SNN training

With the proven robustness of SNN with quantized membrane potential, recovering accuracy degradation becomes a critical task. To counter this issue, we propose a novel quantization algorithm designed for SNN training with low-precision membranes.

Motivated by the *dequantization challenge* in the introduction section, the proposed method quantizes the membrane potential without introducing dequantization scaling (Jacob et al., 2018), leading to high hardware simplicity and efficiency. Unlike prior works (Putra et al., 2022) that incorporated high-precision *scaling* for quantization, we design an integer-only quantization scheme to assign the membrane potential to the nearest integer level directly. Different from Davies et al. (2018) and Putra et al. (2021) which uses high precision *fixed point integer* on membrane potential, our method compresses the membrane using Equation 7 down to **ternary**.


(7) $$Q(u_{t})=\{\begin{array}{ll} -1 & \text{if }u^{t}\leq 0\\ 0 & \text{if }u^{t}\leq 0.5\\ +1 & \text{if }u^{t}\geq 0.5 \end{array}$$

The non-differentiable quantization operation hinders the backward propagation in both spatial and temporal directions, which can be factorized as per Equations 8 and 9:


(8) $$\begin{array}{l} \frac{\partial L}{\partial S_{t}}=\underset{\text{w.r.t Layer}}{\underset{︸}{\frac{\partial L}{\partial S_{t}^{l+1}}\frac{\partial S_{t}^{l+1}}{\partial S_{t}}}}+\underset{\text{w.r.t time step}}{\underset{︸}{\frac{\partial L}{\partial S_{t+1}^{l}}\frac{\partial S_{t+1}^{l}}{\partial S_{t}}}} \end{array}$$

Where


(9) $$\begin{array}{l} \frac{\partial S_{t+1}^{l}}{\partial S_{t}}=\frac{\partial S_{t+1}^{l}}{\partial u_{t+1}^{l}}\frac{\partial u_{t+1}^{l}}{\partial u_{t}^{l}}\frac{\partial u_{t}^{l}}{\partial S_{t}} \end{array}$$

With the proposed quantization scheme, the membrane potential at each time step is updated with low precision based on Equation 10:


(10) $$\begin{array}{l} u_{t+1}^{l}=\tau \times Q\left(u_{t}^{l}\right)+y_{t}^{l} \end{array}$$

The temporal gradient $\partial u_{t+1}^{l}/\partial u_{t}^{l}$ is inaccessible due to the quantization function *Q*(·) which outputs integer levels only. To resolve this issue, we propose **S**tacked **G**radient **S**urrogation (SGS), which approximates the temporal gradient using the sigmoid function during the backward propagation to overcome the non-differentiability of quantization. The choice of Sigmoid function as SGS is empirical and the performance comparison of different surrogation functions is summarized in Table 2. Formally in Equations 11 and 12, we define SGS-based Q^*^ as follows:


(11) $$\begin{array}{l} Q^{*}\left(u_{t}\right)=\overset{K}{\underset{k=1}{\sum }}T\frac{1}{1+e^{-T\left(u_{qt}-s_{k}\right)}}\left(1-\frac{1}{1+e^{-T\left(u_{qt}-s_{k}\right)}}\right) \end{array}$$

(12) $$\begin{array}{l} \;\text{and }s_{k}=\left(k_{i}+k_{i+1}\right)/2 \end{array}$$

Where *T*, *k*, and *s*_*k*_ represent the smoothness, quantization interval, and shift of each surrogation term respectively. With SGS, we have:


(13) $$\begin{array}{l} \partial u_{t+1}^{l}/\partial u_{t}^{l}=\tau Q^{*}\left(u_{t}\right) \end{array}$$

Combining Equations 8–13, we formulate a smooth gradient propagation flow of low-precision membrane potential training. To endorse the performance of the proposed low-precision membrane potential with the stacked surrogate gradient in Quant-SNN, we demonstrate the high memory efficiency achieved with minimal performance degradation against the DVS-CIFAR10 dataset in **Table 5**.

**Table 2:** Training results of VGG-9 model on DVS-CIFAR10 dataset with different SG schemes.

**Architecture**	**Epochs**	**T**	**SGS**	**Accuracy (%)**
VGG-9	200	30	ArcTan	77.81
VGG-9	200	30	Triangle	70.83
VGG-9	200	30	Piece-wise	78.81
VGG-9	200	30	**Sigmoid**	**80.04**

#### 4.2.2 Incorporating weight quantization in SNN training

On top of membrane potential quantization, we incorporate low-precision weights to reduce memory occupancy. To implement complete low-precision inference in Quant-SNN, we adopt and modify the Power of Two (PoT) quantizer (Przewlocka-Rus et al., 2022).


(14) $$\alpha_{w}=(w-1)(argmin_{\alpha }|w-w_{q}|))$$

(15) $$\begin{array}{l} \alpha =round\left(\frac{\alpha_{w}}{2}+\frac{1}{2}\right) \end{array}$$

(16) $$\begin{array}{l} w_{c}=clip\left(round\left(\frac{w}{\alpha }\right),min,max\right) \end{array}$$

In Equation 14–16, *w*, *w*_*q*_, and *w*_*c*_ represent input, quantized and clipped weights. To achieve exact POT weight quantization, we also keep α in the power of two. Subsequently, we draw a POT-based grid *g* in Equation 17 and round the full precision and clipped weight to the nearest bit-map level in Equation 18:


(17) $$g=\{\begin{array}{ll} 0 & \text{if }k\leq 0\\ 2^{-k-1} & \text{if }0<k\leq b \end{array}$$

(18) $$\begin{array}{l} w_{q}=round\left(w_{c}\left[min\left(\left|w_{c}-g\right|\right)\right].\alpha \right) \end{array}$$

We choose 8-bit, 4-bit, and 2-bit precision to compress the layer-wise weights for both object detection and classification tasks. Binary input spikes of each layer convolving the low precision weights can be formulated as approximated computing or look-up tables, which further enhance the hardware efficiency in practice. In the end, we compute the total memory of Quant-SNN and develop a total memory vs. accuracy comparison with the current SOTA in Section 5.

### 4.3 Sparse binary membrane: motivation for sparsity exploration in SNNs

The integer-only membrane exhibits a strong potential for sparsity. Directly ignoring the negative membrane neuron values after quantization shows minimum accuracy degradation. Mathematically, after quantizing the membrane potential with *Q*(·), using Equations 19 and 20 we **disable** the spikes that are generated by the negative membrane values with *U*_*mask*_:


(19) $$\begin{array}{l} U_{mask}=\text{Bool}\left(Q\left(u_{t}\right)<0\right) \end{array}$$

(20) $$\begin{array}{l} S_{t}=S_{t}\left(1-U_{mask}\right) \end{array}$$

In particular, the negative membrane neuron values are “pruned” from the spiking process, which can be skipped during membrane potential updates.

In other words, the membrane potential of the proposed Quant-SNN can be further compressed down to **binary** by sparsifying the original ternary membrane. As shown in Table 3, directly silencing all the “-1” of the membrane shows minimal accuracy degradation **without any fine-tuning**.

**Table 3:** Quant-SNN performance with pruned ternary membrane potential on different datasets.

**Architecture**	**Dataset**	**Binarized Mem**	**Time step**	**Accuracy**
ResNet-19	CIFAR-100	No	2	74.63
ResNet-19	CIFAR-100	Yes	2	74.30
VGG-9	DVS-CIFAR-10	No	2	78.13
VGG-9	DVS-CIFAR-10	Yes	2	77.16
VGG-9	N-Caltech	No	2	80.45
VGG-9	N-Caltech	Yes	2	79.14

The minimum accuracy degradation implies the improved robustness empowered by the sparse and quantized SNN (SpQuant-SNN) training along with pruning opportunities. The pruned binary membrane potential improves the memory efficiency of SpQuant-SNN even further. The low firing rate, high percentage of the negative potential, and robustness of quantized SNN motivate us to explore the membrane potential-based spatial-channel sparsity before the membrane potential accumulation process.

#### 4.3.1 Spatial pruning with membrane potential importance

Following the aforementioned assumptions of low firing rate and a high percentage of negative membrane potential, we skip the membrane potential accumulation for non-significant membrane values before the “accumulate-and-fire” operation. To implement this, we compute the importance score for each time step and apply the pruning threshold using Equations 21 and 22 to mask unwanted spatial membrane values.


(21) $$\begin{array}{l} Imp_{t}^{l}=||Q\left(u_{t}^{l}\right)|{|}_{2} \end{array}$$

(22) $$\begin{array}{l} U_{sm}\;=\;Imp_{t}^{l}>\text{kthvalue}\left(\text{Imp, }Q\left(u_{t}\right)\;\cdot \;sparsity\right),\;U_{t}^{l}\;=\;U_{sm}\;\times \;Q\left(u_{t}^{l}\right) \end{array}$$

For example, we compute the spatial mask $U_{sm}^{l}$ after the first convolution operations at layer “l” and mask out the neuron values at each time step before the accumulate operation. In contrast to post-spike masking, this approach saves redundant additions and skips the weak convolution connections in the subsequent layers. The choice of sparsity value is linked with the robustness of our proposed SpQuant-SNN. Therefore, we mask out 35% neuron values in the spatial domain to maintain the performance along with reduction in computations. We also implement dynamic channel masking to further reduce the compute operations inside SNNs. Channel gating for DNNs has been very well explored in the prior works (Hua et al., 2019; Li et al., 2021a), however, its implementation in SNNs is limited due to the spatial-temporal information propagation. Since SNNs involve time step information for membrane potential accumulation, naive parallel path computation for time step data adds more FLOPs in comparison to the skipped connections. Furthermore, the lottery (Kim et al., 2022) ticket hypothesis does not fit well on the complex architectures and datasets. To address such shortcomings, we propose a straightforward channel-skipping approach, with minimal computational overhead, using membrane potential prior. In the convolution layer of SNNs, $U^{l}\in {\mathbb{R}}^{C_{o}^{l}\times C_{i}^{l}\times T^{l}\times W^{l}\times H^{l}}$ represents the membrane potential feature map of first convolution layer where *T*^*l*^ represents the time step dimension, *C*_*o*_, is the number of output channels, and *C*_*i*_ is the number of input channels per layer. Before the actual convolution for layer *l*+1, we apply a parallel path to compute the attention score of each channel using membrane potential prior.

#### 4.3.2 Channel masking with membrane potential prior

**Figure 3:**
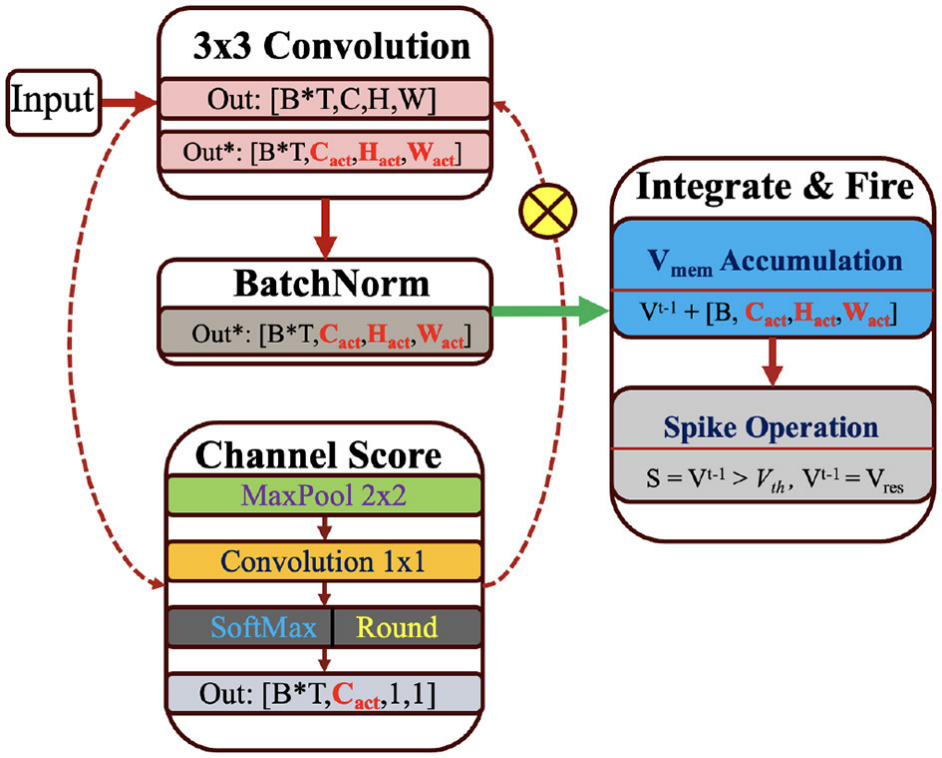
Spatial-channel masking flow of SpQuant-SNN using membrane potential prior.

To achieve low-compute and resource-efficient channel masking, we first pass the $U^{l}\in {\mathbb{R}}^{C_{o}^{l}\times C_{i}^{l}\times W^{l}\times H^{l}}$ through the average pooling layer and extract the succinct spatial attention $U_{mp}^{l}\in {\mathbb{R}}^{C_{o}^{l}\times C_{i}^{l}\times W^{l}/2\times H^{l}/2}$. We consider time-domain pooling by assuming that the first step of spatial pruning leaves salient membrane values across all the time steps. In the second stage, we use point-wise convolution $U_{att}^{l}\in {\mathbb{R}}^{C_{o}^{l+1}\times W^{l}/2\times H^{l}/2}$ to compute the channel-wise attention and sync the out-channels of each input feature map with subsequent layers. To compute the probability score, we use softmax after convolution. Finally, we obtain binary score vector $U_{cm}^{l}\in {\mathbb{R}}^{C_{o}^{l+1}}$ using Equation 23 and apply it as a mask to compress the number of active channels for membrane potential accumulation, as illustrated in Figure 3.


(23) $$U_{sf}^{l}(t)=\frac{e^{U_{att}^{l}}}{\sum_{j=1}^{J}e^{U_{att}^{l}}},\;U_{cm}^{l}=\{\begin{array}{ll} 1 & \text{if }U_{sf}^{l}\geq \theta ,\\ 0 & \text{if }U_{sf}^{l}<\theta . \end{array}$$

To evaluate the impact of spatial-channel pruning, we train SpQuant-VGG9-SNN on the DVS-CIFAR10 dataset. From **Table 5**, SpQuant-SNN achieves 5 × reduction in FLOPs with < 1% accuracy degradation from the quantized membrane and quantized weight SNN baseline. Furthermore, we compare the SpQuant-SNN performance for VGG-9 model with existing full-precision and non-sparse SOTA works and observe a minimal drop in performance with significant resource optimization.

### 4.4 Learning the threshold of membrane potential

To further improve the performance of the proposed SpQuant-SNN, we implement a novel SNN training algorithm that resolves the contradiction between threshold optimization, training stability, and hardware compatibility. We optimize the layer-wise potential threshold during training, maximizing the biological plausibility of SNN without introducing any learning constraints.

Previous works assume that the surrogate gradient of $\frac{\partial S_{t}}{\partial u_{t}}$ and $\frac{\partial S_{t}}{\partial V_{th}}$ is transferrable, and the identical gradient surrogation is suitable for separate loss landscape with respect to *u*_*t*_ and *V*_*th*_. To implement the membrane threshold optimization, we correct the common surrogate landscape assumption and propose a Separate Gradient Path (SGP), which treats the gradient computation of *u*_*t*_ and *V*_*th*_ from Equation 24 with dedicated gradient approximations. We compare the performance of our proposed SGP with the existing threshold optimization technique and validate a better performance based on our proposed SGP optimization in Table 4. Specifically, SGP trains SNNs by introducing the Gradient Penalty Window (GPW), a simple-yet-effective method that optimizes the potential threshold without losing training stability. On top of the gradient approximation in Equation 4, GPW is characterized as a non-linear function **σ**(·), which reshapes the surrogate gradient of the layer-wise potential threshold *V*_*th*_. Mathematically, the GPW-aided separate gradient path is characterized as:


(24) $$\begin{array}{ll} \frac{\partial S_{t}}{\partial u_{t}} & =\theta^{\prime }\left(u_{t}-V_{th}\right)=\max\left(0,1-|u_{t}-V_{th}|\right) \end{array}$$

(25) $$\begin{array}{l} |\frac{\partial S_{t}}{\partial V_{th}}|=\theta^{\prime }\left(u_{t}-V_{th}\right)\sigma \left(u_{t}-V_{th}\right)=\max\left(0,1-|u_{t}-V_{th}|\right)\\ \;\sigma \left(u_{t}-V_{th}\right) \end{array}$$

In this work, we choose the Sigmoid function as the gradient penalty window, presented in Equation 26, for the potential threshold. Here the choice of Sigmoid function is based on the ablation study, presented in Table 2.


(26) $$\begin{array}{l} \sigma \left(u_{t}-V_{th}\right)=\frac{1}{1+e^{-\left(u_{t}-V_{th}\right)}} \end{array}$$

For gradient computation of *V*_*th*_, we accumulate the gradient computed in Equation 25 to avoid the dimensionality mismatch:


(27) $$\frac{\partial L}{\partial V_{th}}=\sum (⊮\{u_{t}\geq V_{th}\}\times |\frac{\partial L}{\partial V_{th}}|)$$

Since the unfired neurons have no contribution to the final loss, the indicator function ⊮{*u*_*t*_≥*V*_*th*_} in Equation 27 only keeps the gradient with respect to the active neurons in the forward pass.

**Table 4:** Performance comparison of SGP with existing SOTA works on DVS-CIFAR10.

**Method**	**Learnable threshold**	**SG function**	**Top-1 accuracy (%)**
TET (Deng et al., 2021)	✗	Triangle	77.33
Penalty for all	✓	GPW for all	76.40
**This work**	✓	**SGP**	**80.04**

We evaluate the performance of SpQuant-SNN with an adaptive threshold using a similar training setup as Quant-SNN. Before implementing the adaptive threshold to SpQuant-SNN directly, we implement it on vanilla SNN and Quant-SNN to properly benchmark the performance. Using adaptive-SNN, we first improve the baseline of vanilla SNN by 1.3% and Quant-SNN by 1.2%. Similarly, from Table 5, applying SpQuant-SNN for VGG-9 model with layer-wise adaptive threshold improves the baseline accuracy by 0.86%.

**Table 5:** Impact of quantization, pruning, and adaptive threshold on SpQuant-SNN performance with VGG-9 model using DVS-CIFAR10 dataset.

**Method**	**Learnable threshold**	**Membrane pot. precision**	**Weight precision**	**Total memory (MB)**	**FLOPs reduction**	**Top-1 accuracy (%)**
TET (Deng et al., 2021)	✗	32-bit	32-bit	48.58	1X	77.33
DSR (Meng et al., 2022)	✗	32-bit	32-bit	48.58	1X	75.50
SNN-Baseline	✗	32-bit	32-bit	48.58	1X	77.20
**Quant-SNN**	✗	**1.58-bit**	**2-bit**	**3.75**	**1X**	**76.84**
Quant-SNN-Baseline	✗	1.58-bit	2-bit	3.75	1X	76.84
**SpQuant-SNN**	✗	**1.58-bit**	**2-bit**	**3.75**	**5X**	**75.96**
Adaptive-SNN-Baseline	✓	32-bit	32-bit	48.58	1X	79.45
**Adaptive-Quant-SNN**	✓	**1.58-bit**	**2-bit**	**3.75**	**1X**	**77.94**
**Adaptive-SpQuant-SNN**	✓	**1.58-bit**	**2-bit**	**3.75**	**5X**	**76.80**

## 5 Experimental results

We validate the proposed SpQuant-SNN algorithm with both event-based and static image computer vision datasets. From event-based datasets, we use DVS-CIFAR10 (Li et al., 2017), N-Cars (Sironi et al., 2018), N-Caltech101 (Orchard et al., 2015) and Prophesee Automotive Gen1 (de Tournemire et al., 2020) to train SpQuant-SNN. Further, we use CIFAR-10 (Krizhevsky et al., 2009), CIFAR-100 (Krizhevsky et al., 2009), ImageNet-100 (Deng et al., 2009), and ImageNet-1k (Deng et al., 2009) datasets for SpQuant-SNN training and inference on static image datasets.

### 5.1 Data preprocessing

The open-sourced DVS datasets are in the shape of indexed bit stream where each chunk represents the axis information, spike polarity, time-step information, and addresses. At the data preprocessing stage, we extract spike polarity and time-step information from the bit streams and convert them to 128x128 binary frames. We sample over different time steps and transform events to 5-D tensors of shape [Batch, Time, Channels, Height, Width]. For static image data including CIFAR-10, CIFAR-100, and ImageNet-100, we use the 8-bit static images for the training and inference (Garg et al., 2021). In the case of static image data (e.g., ImageNet-100), the input shape is in the form of a 5D tensor [N, T, C, H, W], representing batch size, time steps, channel, height, and width, respectively. We repeat the static RGB frames by T times to introduce the temporal domain to the input.

On the other hand, we convert Prophesee Gen1 events to binary histograms by sampling over all the time steps. Then the generated binary histograms are synchronized with artificial ground truth from Perot et al. (2020). Finally, the events and annotations are translated to the tensors of shape [Batch, Time, Channels, Height, Width] and [Batch, Number of boxes, Bounding box] respectively. Unlike prior works (Zhou et al., 2023b), we do not use data augmentation to train SpQuant-SNN for performance improvements.

### 5.2 Experimental setup

For static image-based tasks, we train SpQuant-SNN-based ResNet-19, ResNet-34, and SpikeFomer architectures and characterize the accuracy for CIFAR and ImageNet datasets. For the event-based tasks, we choose the SpQuant-SNN aided VGG-9 and Custom-YOLO-V2 for classification and object detection algorithms on DVS-CIFAR10, N-CalTech101, and Prophesee Gen1 datasets, as shown in the Table 6.

**Table 6:** Model architectures for SpQuant-SNN training.

**Model**	**Architecture**
MobileNet-Light	32C3-64DW-64DW-AP2-128DW- 128DW-AP2-256DW-AP2-FC256-FC10
VGG-7	32C3-32C3-AP2-64C3-64C3-AP2- 128C3-128C3-AP2-256C3-256C3-AP2-FC10
VGG-9	64C3-128C3-AP2-256C3-256C3-AP2- 512C3-512C3-512C3-512C3-AP2-1024C3-AP2-FC10
Custom-Yolo-V2	32C3-MP2-64C3-MP2-128C3-64C1-128C3-MP2- 256C3-128C1-256C3-MP2-512C3-256C1-512C3- 256C1-MP2-1024C3-512C1-1024C3-AP2-FC512-FC576
ResNet-19	64C3-128C3-128C3-128C3-128C3-128C3-128C3- 256C3-256C3-256C3-256C3-256C3-256C3-256C3- 256C3-512C3-512C3-512C3-512C3-AP2-FC256-FC10
ResNet-26	64C3-128C3-128C3-128C3-128C3-128C3-128C3- 256C3-256C3-256C3-256C3-256C3-256C3-256C3- 256C3-512C3-512C3-512C3-512C3-512C3-512C3- 512C3-512C3-512C3-512C3-512C3-512C3-AP2-FC256-FC10

“C3”, “DW”, “MP2”, “AP2” and “FC” represent 3 × 3 convolution layer, 2 × 2 max-pooling, 2 × 2 average-pooling, and fully connected layer.

**Figure 4:**
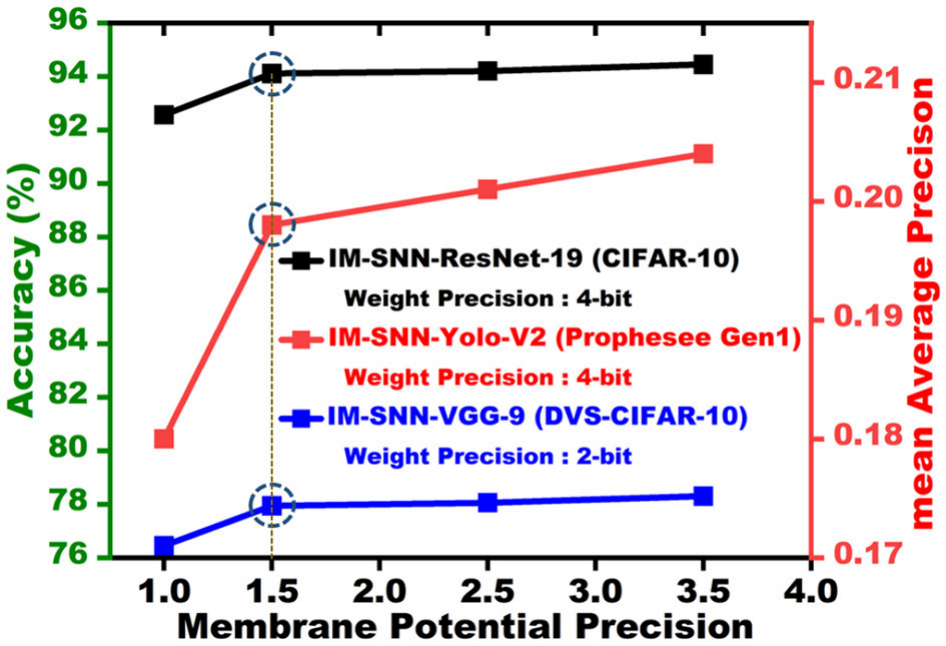
Membrane potential precision vs. accuracy for CIFAR-10 and DVS-CIFAR10 datasets and membrane potential vs. mean average precision for Prophesee Gen1 object detection dataset.

We train our proposed SpQuant-SNN-based classification and object detection architectures using PyTorch (Paszke et al., 2019) version 1.9.0 with CUDA version 11.1. Regarding hyperparameter selection, we use the Adam optimizer where the learning rate is set to 0.001. We computed TET (Deng et al., 2021) loss between the logits and the target labels then compute the average along the time domain. The regularization level β is set to 0.45 and 0.90 for both the full-precision and low-precision training of VGG and ResNet architectures, respectively. Instead of the fixed threshold, we use the learnable threshold in the LIF function for all the classification and object detection networks. With the proposed SpQuant-SNN, we sweep across the different quantization levels to choose the optimal precision, presented in Figure 4, for the membrane potential quantization to achieve high performance with maximum memory reduction across various architectures and datasets. Therefore, considering the performance vs memory trade-off, we optimally quantize the membrane potential to ternary levels [−1.0, 0.0, 1.0] for each time step. In addition to the membrane potential, we quantize the weights to achieve the complete low-precision flow of SNN. We implement 8-bit, 4-bit, and 2-bit weight precision using the APOT quantization scheme (Przewlocka-Rus et al., 2022) for SpQuant-SNN training. Further, to evaluate the pruning opportunities, we choose a 35% pruning ratio in the spatial domain and set the channel masking threshold to 0.47 to balance out the performance with maximum FLOP reduction. To benchmark the performance of the proposed SpQuant-SNN, we compare the Ops (integer-only operations) of SpQuant-SNN with FLOPs of recent SOTA works.

### 5.3 Evaluation metric

We evaluate the SpQuant-SNN based on memory reduction, FLOPs reduction, robustness, and accuracy. We compute the total memory (MB) acquisition of each SpQuant-SNN-based architecture for all the datasets, where the total memory constitutes weight, membrane potential, and convolution output memory:


(28) $$\begin{array}{l} M_{t}=w_{p}\times N_{w}+u_{p}\times N_{f}+u_{s}\times N_{f}\;\text{Where }N_{w}=\overset{\ell }{\underset{l=1}{\sum }}w^{l} \end{array}$$

(29) $$\begin{array}{l} \text{And }N_{f}=\overset{\ell }{\underset{l=1}{\sum }}{\left(C\times W\times H\right)}^{l} \end{array}$$

*M*_*t*_ is the total memory, *w*_*p*_ is weight precision, *u*_*p*_ is membrane potential precision, *u*_*s*_ is the binary spike precision (0,1), *N*_*w*_ is total network weights, and *N*_*f*_ is layer-wise spikes. Further, *w* represents network weights, **ℓ** is the number of layers, *C* is the number of output channels, and *W* and *H* represent the width and height of the input frame.

Furthermore, to evaluate the FLOPs compression in Equation 30 we consider spatially active neurons with active channels and compare them with the conventional SNN flow:


(30) $$\begin{array}{r} FLOPS_{SpQuant-SNN}=2\times C^{l}\left(1-S_{c}\right)\times W\times H\times k^{l}\times k^{l}+\\ \;T\times B\times C^{l}\left(1-S_{c}\right)\times W\times H\times \left(1-S_{p}\right) \end{array}$$

Here *FLOPS*_*SpQuant*−*SNN*_ is the reduced FLOPs, *C*^*l*^, *S*_*c*_, *S*_*p*_, *T*, and *k* represent the out-channels, channel sparsity, spatial sparsity, time step and kernel-size respectively.

### 5.4 RGB/DVS classification

#### 5.4.1 SpQuant-SNN performance on DVS datasets

**Figure 5:**
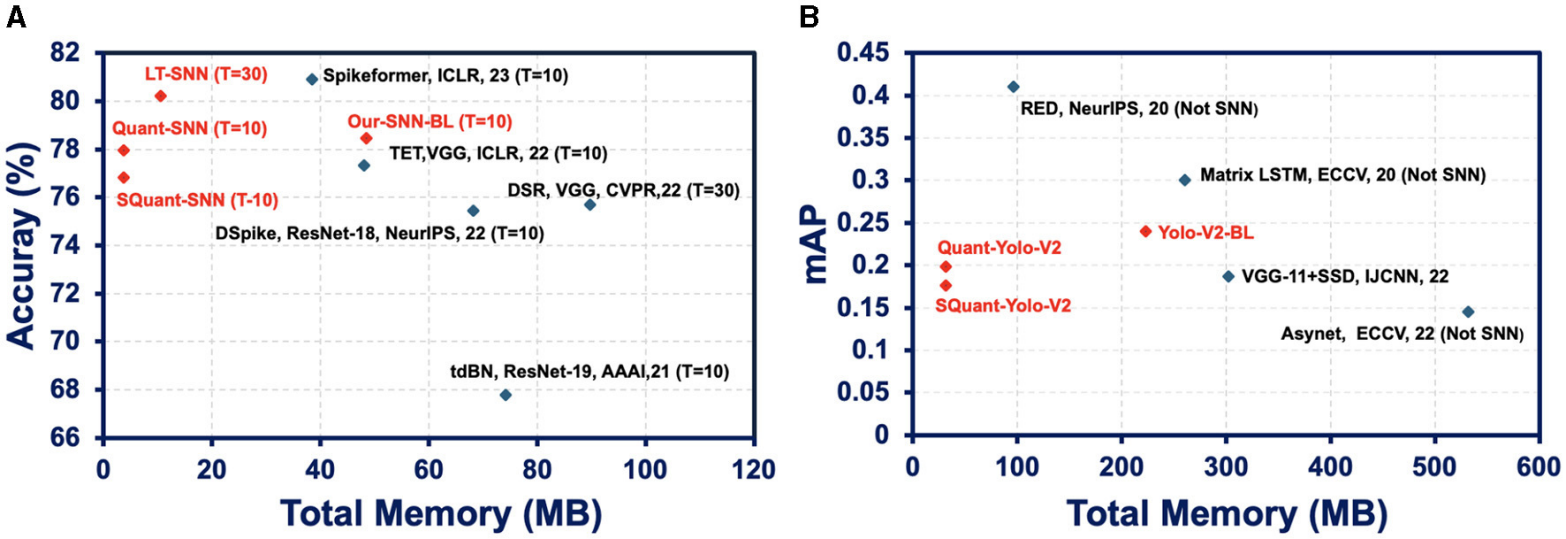
**(A)** Memory and performance comparison of SpQuant-SNN for VGG-9 on DVS-CIFAR10 dataset. **(B)** Memory and performance comparison of SpQuant-SNN for Yolo-V2 on Prophesse Gen 1 dataset.

We compare the performance of sparse quantized SNN SpQuant-SNN with existing SOTA works, implementing full-precision, low-precision SNNs with and without sparse computations. For DVS datasets, we benchmark SpQuant-SNN on DVS-CIFAR10 and N-Caltech for the object classification task. As mentioned in the previous sections, we evaluate the adaptive SpQuant-SNN performance sequentially. We distinguish the impact of quantization and pruning and evaluate low-precision SNN with sparsity (SpQuant-SNN) and without sparsity (Quant-SNN). Table 7 demonstrates the comparison of the proposed algorithm with existing SOTAs in the context of memory efficiency, time step, robustness, FLOPs reduction, and top-1 accuracy. For the DVS dataset, our low-precision adaptive Quant-SNN implementation reduces the memory by 13 × with a 0.51% accuracy drop from the full precision baseline. Furthermore, Quant-SNN with high memory efficiency surpasses the existing convolution-based full-precision counterparts. Finally, with SpQuant-SNN, we attain up to 5 × FLOPs reduction and 13 × memory reduction with an accuracy drop of 1.14% and 1.8% for DVS-CIFAR-10 and N-Caltech datasets. Compared with existing SOTA, the proposed low-precision algorithm, reducing memory occupancy by **13**× and FLOPs by **5**×, almost matches the cutting-edge SNN accuracies for DVS-CIFAR-10 and N-Caltech datasets, respectively. Furthermore, Figure 5A shows the memory and performance comparison of SpQuant-SNN for VGG-9 on DVS-CIFAR10 dataset.

**Table 7:** Experimental results of Quant-SNN and SpQuant-SNN on DVS datasets using T = 10.

**Method**	**Architecture**	**Weight precision**	***U*_*mem*_ precision**	**Weight memory (MB)**	***U*_*mem*_ memory (MB)**	**Total memory (MB)**	**FLOPs reduction**	**Top-1 accuracy**
DVS-CIFAR10								
tdBN (Zheng et al., 2021)	ResNet-19	32-bit	32-bit	49.94	12.09	74.20	1 ×	67.80%
TET (Deng et al., 2021)	VGG-Like	32-bit	32-bit	40.65	3.68	48.01	1 ×	77.33%
DSR (Meng et al., 2022)	VGG-11	32-bit	32-bit	70.43	9.7	89.7	1 ×	75.70%
Dspike (Li et al., 2021b)	ResNet-18	32-bit	32-bit	44.72	11.7	68.12	1 ×	75.45%
Spikformer (Zhou et al., 2023b)	Spikformer-2-256	32-bit	32-bit	38.5	NA	38.5	1 ×	80.90%
Our work (SNN-BL)	VGG-9	32-bit	32-bit	41.12	3.68	48.58	1 ×	78.45%
Our work (Quant-SNN)	VGG-9	2-bit	1.58-bit	2.57	0.23	3.75	1 ×	77.94% (-0.51)
Our work (SpQuant-SNN)	VGG-9	2-bit	1.58-bit	2.57	0.23	3.75	5.0×	76.80% (-1.14)
N-Caltech								
YOLE (Cannici et al., 2019)	VGG7-Like	32-bit	32-bit	42.69	2.01	46.71	1 ×	70.02%
EST (Gehrig et al., 2019)	ResNet-34	32-bit	32-bit	88.39	43.4	175.19	1 ×	78.70%
Asynet (Messikommer et al., 2020)	VGG-13	32-bit	32-bit	22.32	4.09	30.50	1 ×	76.10%
Our work (SNN-BL)	VGG-9	32-bit	32-bit	41.12	3.68	48.58	1 ×	80.45%
Our work (Quant-SNN)	VGG-9	2-bit	1.58-bit	2.57	0.23	3.75	1 ×	79.45% (-1.0)
Our work (SpQuant-SNN)	VGG-9	2-bit	1.58-bit	2.57	0.23	3.75	4.8×	78.65% (-1.8)

#### 5.4.2 SpQuant-SNN performance on static image datasets

Similarly, we evaluate SpQuant-SNN performance on various complex static image datasets. We compare SpQuant-SNN with full-precision and low-precision sparse and non-sparse SOTA SNN works. Identical to the DVS datasets, we evaluate the performance of both Quant-SNN and SpQuant-SNN sequentially. From Table 8, Quant-SNN with low-precision of membrane and weight, yields 8.13 × high memory efficiency with 0.45% and 0.91% drop in performance for both CIFAR-10 and CIFAR-100 datasets. On top of this, along with 8.13 × reduction in memory consumption, SpQuant-SNN achieves up to 5.1 × FLOPs reduction with minimal accuracy degradation of 1.48% and 1.91% from SOTA SNN baseline for CIFAR-10 and CIFAR-100. For Imagenet datasets, Quant-SNN achieves 4.58 × memory reduction with an accuracy drop of 0.34% and 0.64% for ImageNet-100 and ImageNet-1k datasets. Furthermore, SpQuant-SNN achieves 4 × FLOPs and 4.58 × memory reduction with 1.36% and 1.34% accuracy drop from the baseline on the ImageNet-100 and ImageNet-1k datasets.

**Table 8:** Experimental results of SpQuant-SNN on static image datasets including CIFAR-10 with T = 4 and T = 2 (our work), CIFAR-100 with T = 2, ImageNet-100 with T = 2, and Imagenet-1k with T = 4.

**Method**	**Architecture**	**Weight precision**	***U_mem_* precision**	**Weight memory (MB)**	***U_mem_* memory (MB)**	**Total memory (MB)**	**FLOPs reduction**	**Top-1 accuracy**
**CIFAR-10**								
tdBN (Zheng et al., 2021)	ResNet-19	32-bit	32-bit	49.94	5.5	60.94	1 ×	93.16%
DSR (Meng et al., 2022)	ResNet-18	32-bit	32-bit	44.72	5.33	55.38	1 ×	91.89%
Dspike (Li et al., 2021b)	ResNet-18	32-bit	32-bit	44.72	5.33	55.38	1 ×	94.25%
Spikformer (Zhou et al., 2023b)	Spikformer-4-256	32-bit	32-bit	38.20	NA	38.20	1 ×	95.51%
MINT (Yin et al., 2023)	ResNet-19	8-bit	8-bit	12.48	1.37	15.22	1 ×	91.36%
MINT (Yin et al., 2023)	ResNet-19	4-bit	4-bit	6.24	0.69	8.30	1 ×	91.45%
MINT (Yin et al., 2023)	ResNet-19	2-bit	2-bit	3.12	0.35	4.84	1 ×	90.79%
Our work (SNN-BL)	ResNet-19	32-bit	32-bit	49.94	5.5	60.94	1 ×	94.56%
Our work (Quant-SNN)	ResNet-19	4-bit	1.58-bit	6.24	0.25	7.49	1 ×	94.11% (-0.45)
Our work (Quant-SNN)	Spikformer-4-256	8-bit	1.58-bit	9.62	0.25	15.26	1 ×	94.99% (-0.52)
Our work (SpQuant-SNN)	ResNet-19	4-bit	1.58-bit	6.24	0.25	7.49	5.1×	93.09% (-1.48)
CIFAR-100								
DSR (Meng et al., 2022)	ResNet-18	32-bit	32-bit	44.72	5.33	55.38	1 ×	68.33%
TET (Deng et al., 2021)	ResNet-19	32-bit	32-bit	49.94	5.33	60.94	1 ×	72.87%
Our work (SNN-BL)	ResNet-19	32-bit	32-bit	49.94	5.5	60.94	1 ×	72.78%
Our work (Quant-SNN)	ResNet-19	4-bit	1.58-bit	6.24	0.25	7.49	1 ×	71.87% (-0.91)
Our work (SpQuant-SNN)	ResNet-19	4-bit	1.58-bit	6.24	0.25	7.49	4.7×	70.87% (-1.91)
ImageNet-100								
TET (Deng et al., 2021)	ResNet-34	32-bit	32-bit	87.19	11.03	109.25	1 ×	74.70%
Our work (SNN-BL)	ResNet-34	8-bit	1.58-bit	21.27	0.51	23.83	1 ×	74.76%
Our work (Quant-SNN)	ResNet-34	8-bit	1.58-bit	21.27	0.51	23.83	1 ×	74.42% (-0.34)
Our work (SpQuant-SNN)	ResNet-34	8-bit	1.58-bit	21.27	0.51	23.83	4 ×	73.40% (-1.36)
ImageNet-1K								
tdBN (Zheng et al., 2021)	ResNet-34	32-bit	32-bit	87.19	11.03	109.25	1 ×	63.72%
TET (Deng et al., 2021)	ResNet-34	32-bit	32-bit	87.19	11.03	109.25	1 ×	68.00%
Dspike (Li et al., 2021b)	ResNet-34	32-bit	32-bit	87.19	11.03	109.25	1 ×	68.41%
Our work (SNN-BL)	ResNet-34	8-bit	1.58-bit	21.27	0.51	23.83	1 ×	68.46%
Our work (Quant-SNN)	ResNet-34	8-bit	1.58-bit	21.27	0.51	23.83	1 ×	67.82% (-0.64)
Our work (SpQuant-SNN)	ResNet-34	8-bit	1.58-bit	21.27	0.51	23.83	4 ×	67.12% (-1.34)

Finally, we compared the performance of SpQuant-SNN with very recent low-precision SNN works i-e. MINT (Yin et al., 2023). Compared to the MINT-SNN which incorporates low precision membrane potential from 8-bit to 2-bit, the proposed SpQuant-SNN achieves 2.3% higher accuracy together with the 1.58-bit membrane potential and 5.1 × FLOPs reduction with spatial-channel pruning, as shown in Table 8.

### 5.5 Object detection with SpQuant-SNN

We further demonstrate the performance of the proposed SpQuant-SNN on a large-sized Automotive Prohesee Gen1 dataset (de Tournemire et al., 2020). We convert the DVS events to binary frames and synchronized them with their actual ground truths. To avoid the gradient vanishing in SpQuant-SNN-YoloV2, we customize the YoloV2 model by skipping one convolution block from the original architecture. We quantize the membrane potential to ternary levels [−1.0, 0.0, 1.0] just like the classification task and use 4-bit precision for weights to train SpQuant-SNN-Custom-YoloV2. As shown in Table 9, our proposed SpQuant-SNN algorithm with custom-YoloV2 reduces memory utilization by 7.07 × and FLOPs by 4.7 × in comparison to the full-precision baseline with a decrement of 0.042 in the mAP against Prophesee Gen1 dataset. Furthermore, we illustrate the memory vs. mAP performance comparison of SpQuant-SNN-YoloV2 in Figure 5B.

**Table 9:** Experimental results of the proposed SpQuant-SNN on Prophesee Automotive Gen1 dataset.

**Dataset**	**Method**	**SNN**	**Weight precision**	***U_mem_ precision***	**Total memory (MB)**	**FLOPs reduction**	**mAP**
Asynet (Messikommer et al., 2020)	FB-Dense	No	32	-	532.00	1 ×	0.145
MatrixLSTM (Cannici et al., 2020)	ResNet-19	No	32	-	260.00	1 ×	0.300
RED (Perot et al., 2020)	RetinaNet	No	32	-	96.00	1 ×	0.410
VGG-11+SSD (Cordone et al., 2022)	VGG+SSD-SNN	Yes	32	32	302.39	1 ×	0.187
This work (SNN-BL)	Custom-YoloV2-SNN	Yes	32	32	223.20	1 ×	0.240
This work (Quant-SNN)	Custom-YoloV2-SNN	Yes	4	1.58	31.57	1 ×	0.198
This work (SpQuant-SNN)	Custom-YoloV2-SNN	Yes	4	1.58	31.57	4.7×	0.176

### 5.6 Theoretical energy consumption of SpQuant-SNN

Finally, we compute the theoretical energy consumption of our proposed SpQuant-SNN algorithm for different architectures. We follow BitNet (Wang et al., 2023) to compute the theoretical energy of our proposed work. Since we perform the training on NVIDIA A6000 GPUs, we adopt 7nm energy for add and multiplication operation from Table 2 of BitNet (Wang et al., 2023). Overall, we use Equation 31 to compute the theoretical energy consumption:


(31) $$\begin{array}{l} Theoretical\;E_{SpQuant-SNN}=\\ E_{mul}\times C^{l}\left(1-S_{c}\right)\times W\times H\times k^{l}\times k^{l}+\\ E_{add}\times C^{l}\left(1-S_{c}\right)\times W\times H\times k^{l}\times k^{l}+\\ \;E_{add}\times T\times B\times C^{l}\left(1-S_{c}\right)\times W\times H\times \left(1-S_{p}\right) \end{array}$$

Table 10 demonstrates the theoretical energy consumption of our proposed SqQuant-SNN-based architectures along with their performance. Furthermore, we observe that Spikformer (Zhou et al., 2023b) and BitNet (Wang et al., 2023) do consider the membrane potential accumulation in their energy modeling. For a fair comparison of energy consumption between SpQuant-SNN and Spikformer, it is important to count the membrane potential accumulation energy.

**Table 10:** Theoretical energy consumption vs. performance of SpQuant-SNN with different architectures.

**Architecture**	**Dataset**	**Weight precision**	**Membrane pot. precision**	**E_mul (uJ) theoretical**	**E_add (uJ) theoretical**	**E_total (uJ) theoretical**	**T**	**Top-1 accuracy (%)**
SpQuant-SNN-VGG9	DVS-CIFAR-10	2-bit	1.58-bit	0.29	0.156	0.45	10	76.80
SpQuant-SNN-ResNet19	CIFAR-10	4-bit	1.58-bit	1.04	0.58	1.62	4	93.09
SpQuant-SNN-ResNet34	ImageNet-1k	8-bit	1.58-bit	5.54	2.62	8.16	4	67.12
SpQuant-SNN-Yolov2	Prophesee-Gen1	4-bit	1.58-bit	4.63	2.362	6.99	1	0.176

## 6 Conclusion

In this paper, we propose SpQuant-SNN, a novel SNN algorithm that implements both quantization and pruning to achieve highly efficient SNN with double compression. The integer-only SNN with high sparsity largely reduces memory and compute complexity. The proposed algorithm successfully compresses the membrane potential down to ternary representation, achieving up to 13 × memory footprint reduction, while maintaining the high simplicity of SNN. Furthermore, SpQuant-SNN shows strong robustness in dynamic membrane pruning. SpQuant-SNN, learning the membrane potential prior, implements spatial-channel pruning and achieves >4.7 × reduction in FLOPs. SpQuant-SNN is evaluated on a comprehensive spectrum of computer vision tasks, including both static image classification and event-based object detection. The outstanding versatility makes the proposed SpQuant-SNN a powerful solution for energy-efficient on-device computer vision.

## Data Availability

The original contributions presented in the study are included in the article/supplementary material, further inquiries can be directed to the corresponding author.

## References

[B1] Cannici M. Ciccone M. Romanoni A. Matteucci M. (2019). “Asynchronous convolutional networks for object detection in neuromorphic cameras,” in IEEE/CVF Conference on Computer Vision and Pattern Recognition (CVPR) Workshops (Long Beach, CA: IEEE).

[B2] Cannici M. Ciccone M. Romanoni A. Matteucci M. (2020). “A differentiable recurrent surface for asynchronous event-based data,” in European Conference on Computer Vision (ECCV) (Europe: IEEE Computer Society and Computer Vision Foundation), 136–152.

[B3] Castagnetti A. Pegatoquet A. Miramond B. (2023). Trainable quantization for speedy spiking neural networks. Front. Neurosci. 17:1154241. 10.3389/fnins.2023.1154241 36937675 PMC10020579

[B4] Che K. Leng L. Zhang K. Zhang J. Meng Q. Cheng J.. (2022). “Differentiable hierarchical and surrogate gradient search for spiking neural networks,” in Advances in Neural Information Processing Systems (NeurIPS), 35.

[B5] Chen R. Ma H. Xie S. Guo P. Li P. Wang D. (2018). “Fast and efficient deep sparse multi-strength spiking neural networks with dynamic pruning,” in 2018 International Joint Conference on Neural Networks (IJCNN) (Rio de Janeiro: IEEE), 1-8.

[B6] Chen Y. Zhang S. Ren S. Qu H. (2022). “Gradual surrogate gradient learning in deep spiking neural networks,” in IEEE International Conference on Acoustics, Speech and Signal Processing (ICASSP) (Singapore: IEEE), 8927–8931.

[B7] Chowdhury S. S. Garg I. Roy K. (2021). “Spatio-temporal pruning and quantization for low-latency spiking neural networks,” in International Joint Conference on Neural Networks (IJCNN).

[B8] Cordone L. Miramond B. Thierion P. (2022). Object detection with spiking neural networks on automotive event data. arXiv [Preprint]. arXiv:2205.04339. 10.1109/IJCNN55064.2022.9892618

[B9] Davies M. Srinivas N. Lin T. Chinya G. Cao Y. Choday S. (2018). Loihi: a neuromorphic manycore processor with on-chip learning. IEEE Micro 38, 82–99. 10.1109/MM.2018.112130359

[B10] de Tournemire P. Nitti D. Perot E. Migliore D. Sironi A. (2020). A large scale event-based detection dataset for automotive. arXiv [Preprint]. arXiv:2001.08499.33162883

[B11] Deng S. Gu S. (2021). Optimal conversion of conventional artificial neural networks to spiking neural networks. arXiv [Preprint]. arXiv:2103.00476. 10.48550/arXiv.2103.0047638400487

[B12] Deng S. Li Y. Zhang S. Gu S. (2021). “Temporal efficient training of spiking neural network via gradient re-weighting,” in International Conference on Learning Representations (ICLR).

[B13] Deng J. Dong W. Socher R. Li L.-J. Li K. Fei-Fei L. (2009). “Imagenet: a large-scale hierarchical image database,” in 2009 IEEE Conference on Computer Vision and Pattern Recognition (Miami, FL: IEEE), 248–255.

[B14] Diehl P. U. Neil D. Binas J. Cook M. Liu S.-C. Pfeiffer M. (2015). “Fast-classifying, high-accuracy spiking deep networks through weight and threshold balancing,” in International Joint Conference on Neural Networks (IJCNN), 1–8.36968504

[B15] Ding J. Yu Z. Tian Y. Huang T. (2021). Optimal ann-snn conversion for fast and accurate inference in deep spiking neural networks. arXiv [Preprint]. arXiv:2105.11654. 10.24963/ijcai.2021/321

[B16] Fang W. Yu Z. Chen Y. Masquelier T. Huang T. Tian Y. (2021). “Incorporating learnable membrane time constant to enhance learning of spiking neural networks,” in IEEE/CVF Conference on Computer Vision and Pattern Recognition (CVPR) (Montreal, QC: IEEE), 2661–2671.

[B17] Gallego G. Delbrück T. Orchard G. Bartolozzi C. Taba B. Censi A.. (2020). Event-based vision: a survey. IEEE Trans. Pattern Anal. Mach. Intell. 44, 154–180. 10.1109/TPAMI.2020.3008413 32750812

[B18] Garg I. Chowdhury S. S. Roy K. (2021). “DCT-SNN: Using dct to distribute spatial information over time for low-latency spiking neural networks,” in IEEE/CVF International Conference on Computer Vision (ICCV (Europe: IEEE Computer Society and Computer Vision Foundation).

[B19] Gehrig D. Loquercio A. Derpanis K. G. Scaramuzza D. (2019). “End-to-end learning of representations for asynchronous event-based data,” in IEEE/CVF Conference on Computer Vision and Pattern Recognition (CVPR) (North America: IEEE Computer Society and Computer Vision Foundation), 5633–5643.

[B20] Guo Y. Tong X. Chen Y. Zhang L. Liu X. Ma Z.. (2022). “RecDis-SNN: rectifying membrane potential distribution for directly training spiking neural networks,” in IEEE/CVF Conference on Computer Vision and Pattern Recognition (CVPR) (New Orleans, LA: IEEE), 326–335.

[B21] Han B. Roy K. (2020). “Deep spiking neural network: energy efficiency through time based coding,” in European Conference on Computer Vision (Cham: Springer), 388–404.

[B22] Han B. Srinivasan G. Roy K. (2020). “RMP-SNN: residual membrane potential neuron for enabling deeper high-accuracy and low-latency spiking neural network,” in IEEE/CVF Conference on Computer Vision and Pattern Recognition (CVPR) (Seattle, WA: IEEE), 13558–13567.

[B23] He K. Zhang X. Ren S. Sun J. (2016). “Deep residual learning for image recognition,” in IEEE/CVF Conference on Computer Vision and Pattern Recognition (CVPR) (Las Vegas, NV: IEEE), 770–778.

[B24] Hua W. Zhou Y. De Sa C. Zhang Z. Suh G. E. (2019). “Boosting the performance of cnn accelerators with dynamic fine-grained channel gating,” in Proceedings of the 52nd Annual IEEE/ACM International Symposium on Microarchitecture, 139–150.

[B25] Jacob B. Kligys S. Chen B. Zhu M. Tang M. Howard A.. (2018). “Quantization and training of neural networks for efficient integer-arithmetic-only inference,” in IEEE/CVF Conference on Computer Vision and Pattern Recognition (CVPR) (Salt Lake City, UT: IEEE), 2704–2713.

[B26] Kim Y. Li Y. Park H. Venkatesha Y. Yin R. Panda P. (2022). “Exploring lottery ticket hypothesis in spiking neural networks,” in European Conference on Computer Vision (ECCV) (Cham: Springer).

[B27] Kole M. H. P. Stuart G. J. (2008). Is action potential threshold lowest in the axon? Nat. Neurosci. 11, 1253–1255. 10.1038/nn.2203 18836442

[B28] Krizhevsky A. (2009). Learning Multiple Layers of Features from Tiny Images.

[B29] Lee J. H. Delbruck T. Pfeiffer M. (2016). Training deep spiking neural networks using backpropagation. Front. Neurosci. 10:508. 10.3389/fnins.2016.00508 27877107 PMC5099523

[B30] Li C. Ma L. Furber S. (2022). Quantization framework for fast spiking neural networks. Front. Neurosci. 16:918793. 10.3389/fnins.2022.918793 35928011 PMC9344889

[B31] Li F. Li G. He X. Cheng J. (2021a). “Dynamic dual gating neural networks,” in Proceedings of the IEEE/CVF International Conference on Computer Vision (Montreal, QC: IEEE), 5330–5339.

[B32] Li H. Liu H. Ji X. Li G. Shi L. (2017). Cifar10-DVS: an event-stream dataset for object classification. Front. Neurosci. 11:309. 10.3389/fnins.2017.00309 28611582 PMC5447775

[B33] Li Y. Guo Y. Zhang S. Deng S. Hai Y. Gu S. (2021b). Differentiable spike: rethinking gradient-descent for training spiking neural networks. Adv. Neural Inform. Proc. Syst. 34, 23426–23439.

[B34] Lien H.-H. Chang T.-S. (2022). Sparse compressed spiking neural network accelerator for object detection. IEEE Trans. Circuits Syst. I: Regular Papers 69, 2060–2069. 10.1109/TCSI.2022.3149006

[B35] Meng Q. Xiao M. Yan S. Wang Y. Lin Z. Luo Z.-Q. (2022). “Training high-performance low-latency spiking neural networks by differentiation on spike representation,” in IEEE/CVF Conference on Computer Vision and Pattern Recognition (CVPR) (North America: IEEE Computer Society and Computer Vision Foundation), 12444–12453.

[B36] Messikommer N. Gehrig D. Loquercio A. Scaramuzza D. (2020). “Event-based asynchronous sparse convolutional networks,” in European Conference on Computer Vision (ECCV) (Europe: IEEE Computer Society and Computer Vision Foundation), 415–431.

[B37] Neftci E. O. Mostafa H. Zenke F. (2019). Surrogate gradient learning in spiking neural networks: bringing the power of gradient-based optimization to spiking neural networks. IEEE Signal Process. Mag. 36, 51–63. 10.1109/MSP.2019.2931595

[B38] Orchard G. Jayawant A. Cohen G. K. Thakor N. (2015). Converting static image datasets to spiking neuromorphic datasets using saccades. Front. Neurosci. 9:437. 10.3389/fnins.2015.00437 26635513 PMC4644806

[B39] Park E. Yoo S. (2020). “Profit: A novel training method for sub-4-bit mobilenet models,” in European Conference on Computer Vision (ECCV) (Cham: Springer).

[B40] Paszke A. Gross S. Massa F. Lerer A. Bradbury J. Chanan G.. (2019). “Pytorch: an imperative style, high-performance deep learning library,” in Advances in Neural Information Processing Systems 32 (Red Hook, NY: Curran Associates, Inc), 8024–8035.

[B41] Perez-Nieves N. Goodman D. (2021). Sparse spiking gradient descent. Adv. Neural Inf. Process. Syst. 34, 11795–11808.

[B42] Perot E. de Tournemire P. Nitti D. Masci J. Sironi A. (2020). Learning to detect objects with a 1 megapixel event camera. Adv. Neural Inf. Process. Syst (NeurIPS) 33, 16639–16652.

[B43] Przewlocka-Rus D. Sarwar S. S. Sumbul H. E. Li Y. De Salvo B. (2022). Power-of-two quantization for low bitwidth and hardware compliant neural networks. arXiv [Preprint]. arXiv:2203.05025.

[B44] Putra R. Shafique M. (2021). “Q-spinn: A framework for quantizing spiking neural networks,” in International Joint Conference on Neural Networks (IJCNN) (IEEE).

[B45] Putra R. Shafique M. (2022). tinySNN: Towards memory-and energy-efficient spiking neural networks. arXiv [Preprint]. arXiv:2206.08656.

[B46] Rueckauer B. Lungu I.-A. Hu Y. Pfeiffer M. (2016). Theory and tools for the conversion of analog to spiking convolutional neural networks. arXiv [Preprint]. arXiv:1612.04052.

[B47] Schaefer C. J. Taheri P. Horeni M. Joshi S. (2023). The hardware impact of quantization and pruning for weights in spiking neural networks. IEEE Trans. Circuits Syst. II. 70, 1789–1793. 10.1109/TCSII.2023.3260701

[B48] Sengupta A. Ye Y. Wang R. Liu C. Roy K. (2019). Going deeper in spiking neural networks: VGG and residual architectures. Front. Neurosci. 13:95. 10.3389/fnins.2019.00095 30899212 PMC6416793

[B49] Shen G. Zhao D. Zeng Y. (2022). Backpropagation with biologically plausible spatiotemporal adjustment for training deep spiking neural networks. Patterns 2022:100522. 10.1016/j.patter.2022.100522 35755868 PMC9214320

[B50] Sironi A. Brambilla M. Bourdis N. Lagorce X. Benosman R. (2018). “HATS: histograms of averaged time surfaces for robust event-based object classification,” in IEEE/CVF Conference on Computer Vision and Pattern Recognition (CVPR) (Salt Lake City, UT: IEEE), 1731–1740.

[B51] Wang S. Cheng T. H. Lim M.-H. (2022). Ltmd: Learning improvement of spiking neural networks with learnable thresholding neurons and moderate dropout. Adv. Neural Inf. Process. Syst. 35, 28350–28362.

[B52] Wang H. Ma S. Dong L. Huang S. Wang H. Ma L.. (2023). Bitnet: Scaling 1-bit transformers for large language models. arXiv [Preprint]. arXiv:2310.11453.

[B53] Wu Y. Deng L. Li G. Zhu J. Xie Y. Shi L. (2019). “Direct training for spiking neural networks: faster, larger, better,” in Proceedings of the AAAI Conference on Artificial Intelligence (AAAI), 1311–1318.

[B54] Yin R. Li Y. Moitra A. Panda P. (2023). Mint: Multiplier-less integer quantization for spiking neural networks. arXiv [Preprint]. arXiv:2305.09850. 10.1109/ASP-DAC58780.2024.10473825

[B55] Zheng H. Wu Y. Deng L. Hu Y. Li G. (2021). “Going deeper with directly-trained larger spiking neural networks,” in Proceedings of the AAAI Conference on Artificial Intelligence (AAAI), 11062–11070.

[B56] Zhou Z. Zhu Y. He C. Wang Y. YAN S. Tian Y.. (2023a). “Spikformer: When spiking neural network meets transformer,” in The Eleventh International Conference on Learning Representations.

[B57] Zhou Z. Zhu Y. He C. Wang Y. Yan S. Tian Y.. (2023b). “Spikformer: When spiking neural network meets transformer,” in International Conference on Learning Representations (ICLR).

